# Efficacy and safety of artemether-lumefantrine for the treatment of uncomplicated falciparum malaria in mainland Tanzania, 2018

**DOI:** 10.1186/s12936-024-04926-x

**Published:** 2024-04-06

**Authors:** Billy Ngasala, Mercy G. Chiduo, Samwel Bushukatale, Bruno P. Mmbando, Twilumba Makene, Erasmus Kamugisha, Maimuna Ahmed, Celine I. Mandara, Filbert Francis, Muhidin K. Mahende, Reginald A. Kavishe, Florida Muro, Deus S. Ishengoma, Renata Mandike, Fabrizio Molteni, Frank Chacky, Chonge Kitojo, George Greer, Dunstan Bishanga, Jasmine Chadewa, Ritha Njau, Marian Warsame, Bilali Kabula, Ssanyu S. Nyinondi, Erik Reaves, Ally Mohamed

**Affiliations:** 1 Department of Parasitology, Muhimbili University of Health and Allied Sciences, P.O. Box 65011, Dar es Salaam, Tanzania; 2 Tanga Research Centre, National Institute for Medical Research, P.O Box 5004, Tanga, Tanzania; 3 Catholic University of Health and Allied Sciences/Bugando Medical Centre, P. O Box 1464, Mwanza, Tanzania; 4 National Institute for Medical Research, Headquarters, P.O. Box 9653, Dar-es-Salaam, Tanzania; 5 Ifakara Health Institute Dar es Salaam Office, P. O. Box 78373, Dar es Salaam, Tanzania; 6 Kilimanjaro Christian Medical Centre, P.O. Box 3010, Moshi, Tanzania; 7 National Malaria Control Programme (NMCP), P.O. Box 743, Dodoma, Tanzania; 8 U.S. President’s Malaria Initiative, U.S. Agency for International Development, Dar es Salaam, Tanzania; 9 Department of Community Health, Muhimbili University of Health and Allied Sciences, P.O. Box 65011, Dar es Salaam, Tanzania; 10 Jhpiego, Boresha Afya, P.O. Box 9170, Dar es Salaam, Tanzania; 11 World Health Organization Country Office, P.O Box 9292, Dar es Salaam, Tanzania; 12 Gothenburg University, Gothenburg, Sweden; 13 RTI International, Dar es Salaam, Tanzania; 14 U.S. President’s Malaria Initiative, U.S. Centers for Disease Control and Prevention, Dar es Salaam, Tanzania

**Keywords:** Malaria, *Plasmodium falciparum*, Artemether lumefantrine, Therapeutic efficacy

## Abstract

**Background:**

The use of artemisinin-based combination therapy (ACT) is recommended by the World Health Organization for the treatment of uncomplicated falciparum malaria. Artemether-lumefantrine (AL) is the most widely adopted first-line ACT for uncomplicated malaria in sub-Saharan Africa (SSA), including mainland Tanzania, where it was introduced in December 2006. The WHO recommends regular assessment to monitor the efficacy of the first-line treatment specifically considering that artemisinin partial resistance was reported in Greater Mekong sub-region and has been confirmed in East Africa (Rwanda and Uganda). The main aim of this study was to assess the efficacy and safety of AL for the treatment of uncomplicated falciparum malaria in mainland Tanzania.

**Methods:**

A single-arm prospective anti-malarial drug efficacy trial was conducted in Kibaha, Mlimba, Mkuzi, and Ujiji (in Pwani, Morogoro, Tanga, and Kigoma regions, respectively) in 2018. The sample size of 88 patients per site was determined based on WHO 2009 standard protocol. Participants were febrile patients (documented axillary temperature ≥ 37.5 °C and/or history of fever during the past 24 h) aged 6 months to 10 years**.** Patients received a 6-dose AL regimen by weight twice a day for 3 days. Clinical and parasitological parameters were monitored during 28 days of follow-up to evaluate the drug efficacy and safety.

**Results:**

A total of 653 children were screened for uncomplicated malaria and 349 (53.7%) were enrolled between April and August 2018. Of the enrolled children, 345 (98.9%) completed the 28 days of follow-up or attained the treatment outcomes. There were no early treatment failures, but recurrent infections were higher in Mkuzi (35.2%) and Ujiji (23%). By Kaplan–Meier analysis of polymerase chain reaction (PCR) uncorrected adequate clinical and parasitological response (ACPR) ranged from 63.4% in Mkuzi to 85.9% in Mlimba, while PCR-corrected ACPR on day 28 varied from 97.6% in Ujiji to 100% in Mlimba. The drug was well tolerated; the commonly reported adverse events were cough, runny nose, and abdominal pain. No serious adverse event was reported.

**Conclusion:**

This study showed that AL had adequate efficacy and safety for the treatment of uncomplicated falciparum malaria. The high number of recurrent infections were mainly due to new infections, indicating the necessity of utilizing alternative artemisinin-based combinations, such as artesunate amodiaquine, which provide a significantly longer post-treatment prophylactic effect.

**Supplementary Information:**

The online version contains supplementary material available at 10.1186/s12936-024-04926-x.

## Background

*Plasmodium falciparum* malaria is a major public health threat especially to children under-five years of age and pregnant women in sub-Saharan Africa (SSA). Early diagnosis and prompt treatment with effective drugs remains the mainstay of malaria control to decrease the risk of serious disease and death. One of the major problems in case management is widespread *P. falciparum* resistance to conventional anti-malarials, such as chloroquine and sulfadoxine-pyrimethamine [1, 2].

To mitigate the development of resistance to monotherapy, many malaria endemic countries have adopted and implemented the World Health Organization (WHO) recommendation of introducing artemisinin-based combination therapy(ACT) [3]. Artemisinin-based combinations have been shown to be highly effective and safe against multidrug-resistant *Plasmodium falciparum* [4–6]. Artemether-lumefantrine (AL) is presently the most widely adopted artemisinin-based combination in Africa, and is the recommended first-line treatment for uncomplicated falciparum malaria in mainland Tanzania [7].

Despite high cure rates achieved after treatment with AL, previous studies have shown that both children and adults who are administered ‘unsupervised’ AL achieve significantly lower day-7 lumefantrine concentrations [7–9], [8–11]. This is a major concern, which may compromise future treatment outcome, since low lumefantrine concentrations in combination with the long elimination half-life of lumefantrine may facilitate selection and spread of tolerant/resistant parasites [10–16]. Resistance can also develop to artemisinin derivatives, especially if these drugs are used inappropriately as monotherapies, or through unregulated access to ACT in public and private sectors and community-based delivery systems. Recent reports from Southeast Asia, Rwanda, Uganda, and Eritrea suggest that falciparum parasites now show early signs of resistance to artemisinin-based combinations and to artemisinin monotherapies, characterized by slow parasite clearance i*n vivo* [12–14]. Additionally, findings from Kagera region near the Rwandan border show evidence of artemisinin partial resistance (Ishengoma DS, unpublished data) [17, 18].

In line with the WHO recommendation to monitor the therapeutic efficacy and safety of nationally recommended ACT at least every two years[15], the present study was conducted in 2018 to assess the efficacy and safety of AL for the treatment of uncomplicated *P. falciparum* malaria in children aged six months to 10 years in Mainland Tanzania.

## Methods

### Study sites

**Fig. 1:**
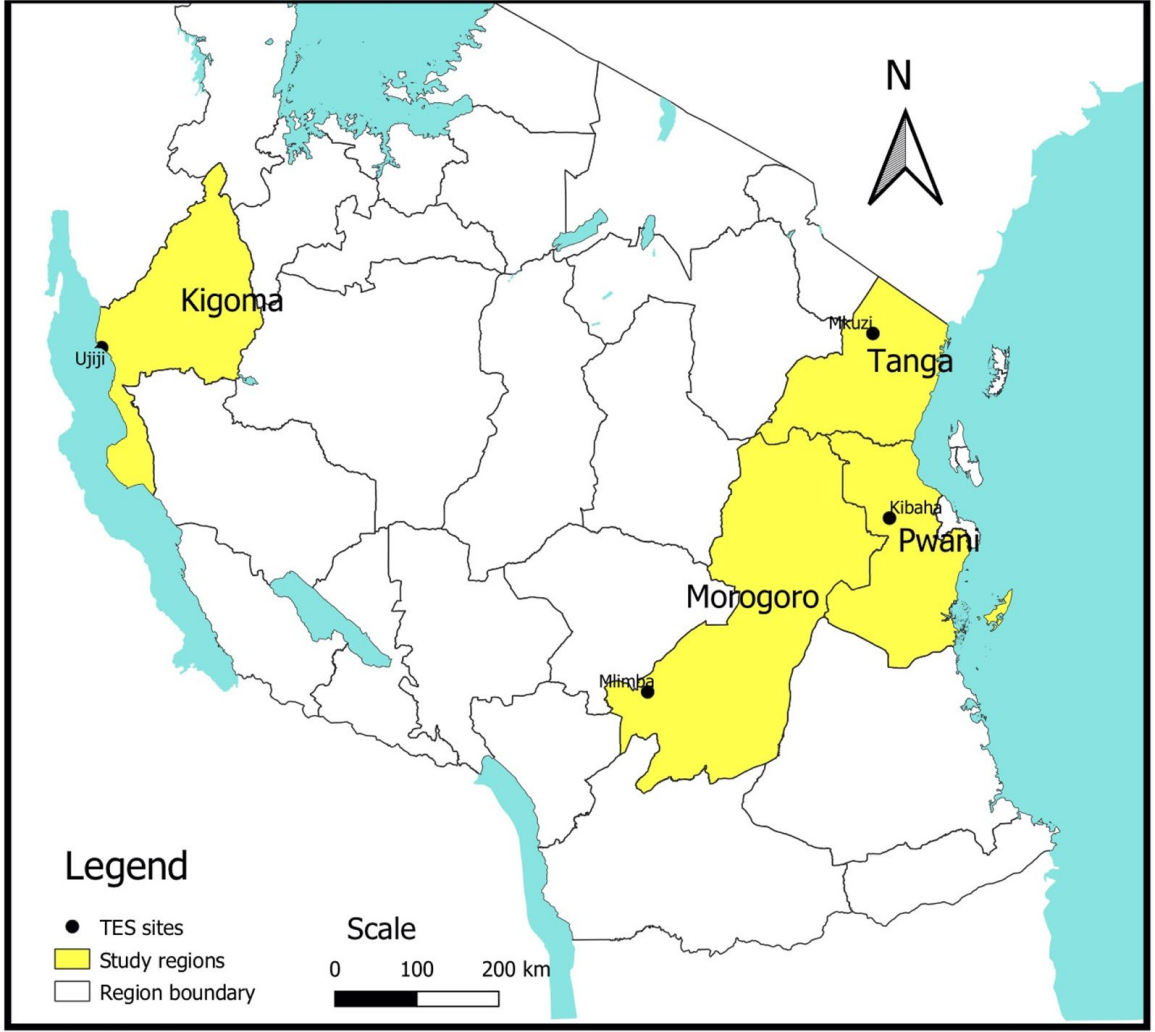
Map of Tanzania showing the study sites by region

The study was carried out in four health facilities across four Regions: Kibaha-Pwani, Mlimba- Morogoro, Mkuzi-Tanga, and Ujiji-Kigoma between April and August 2018 (Fig. 1). These facilities are among the eight National Malaria Control Programme (NMCP) sentinel sites for monitoring therapeutic efficacy of anti-malarial drugs since 1997 [16, 17]. In general, there is high to low malaria transmission in most parts of Mainland Tanzania. However, seasonal transmission peaks occur after the main long rainfall season between June and August with tendencies to shift between March and May in most areas depending on the timing of rainfalls. Throughout Tanzania, *P. falciparum* is the predominant malaria species and *Anopheles gambiae *sensu lato and *Anopheles funestus* are considered the main vectors.

### Study design and participants

This was a single-arm prospective study for assessing the therapeutic efficacy and safety of AL for treatment of uncomplicated falciparum malaria according to the standard WHO protocol [15].

Study participants were recruited among patients presenting at the study sites for care. Inclusion criteria were as follows; children aged between six months and 10 years, fever (axillary temperature ≥ 37.5 °C) and/or reported history of fever in the past 24 h, mono-infection of *P. falciparum* detected by microscopy, parasitaemia between 250 and 200,000 asexual parasites per microlitre of blood, ability to swallow oral medications; ability and willingness to attend scheduled follow-up visits, informed consent provided by parent or guardian, and stable residence within the catchment area throughout the study period.

Initial screening utilized malaria rapid diagnostic tests (mRDTs) followed by microscopy to confirm eligibility of study participants. Exclusion criteria included patients with negative mRDT results and general danger signs or signs of severe falciparum malaria. Patients with mixed or mono-infections with another *Plasmodium* species, severe anaemia (Haemoglobin < 5 g/dL), presence of severe malnutrition (defined as a child who had symmetrical oedema involving at least the feet or mid-upper arm circumference < 110 mm), were excluded from the study. Other exclusion criteria included febrile conditions due to diseases other than malaria (e.g. measles, acute lower respiratory tract infection, severe diarrhoea with dehydration) or other known underlying chronic or severe diseases (e.g. cardiac, renal, and hepatic diseases, HIV/AIDS), regular medications which may interfere with anti-malarial pharmacokinetics, and history of hypersensitivity reactions or contraindications to any of the medicine(s) tested or used as alternative treatment(s). Excluded patients received appropriate treatment according to the national guidelines [18]

### Sample size estimation

The sample size was determined based on WHO 2009 standard protocol [15], with the assumption that 5% of the patients treated with AL were likely to have treatment failure. At a confidence level of 95% and an estimate precision of 5%; a minimum sample size was 73 patients at each site. With 20% increase to allow for the loss to follow-up and withdrawals during the 28-Day follow-up, 88 patients were targeted per site.

### Treatment, laboratory procedures and follow-up

Patients enrolled in the study were treated with artemether-lumefantrine (AL, Coartem®, Beijing Novartis Pharma Ltd, Beijing China for Novartis Pharma AG, Basel, Switzerland) obtained from WHO. This was a fixed combination of 20 mg of artemether and 120 mg lumefantrine in a tablet. The drugs were administered according to the recommended doses based on the weight of patient [3, 18]. One tablet was given to children weighing 5-14 kg; two tablets to children weighing 15–24 kg and three tablets to children weighing 25–35 kg. A full course of AL consisted of 6-doses given twice daily (8 hourly apart on day 0 and 12 hourly apart on days 1 and 2). Food was not provided with treatment; however, guardians were counselled to provide fatty food at home to optimise absorption of the drug. Patients were observed for 30 min to ensure that they did not vomit the study drugs. When vomiting occurred, a repeat dose was given after vomiting stopped. Any patient who persistently vomited the study medication was withdrawn and treated with parenteral quinine or injectable artesunate according to the national guidelines for management of complicated and severe malaria [18]. Paracetamol was given to all patients with body temperature greater than or equal to 38°C. All doses were administered orally under direct observation of a study nurse. Patients with persistent asexual parasitaemia on day 7 or who develop a recurrent parasitaemia after day 7 with no signs of severity were treated with an alternative ACT, either artesunate amodiaquine (ASAQ) or dihydroartemisinin-piperaquine (DHAPQ). In case of development of any danger signs or signs of severe malaria at any point, rescue treatment consisted of parenteral artesunate was given and patients were withdrawn in the study. The procedures for blood smear staining, parasite counting, parasite density calculation, and quality control of blood slide readings are described in the WHO protocol [15]

### Patient follow-up

Follow-up was done for 28 days with scheduled visits on days 1, 2, 3, 7, 14, 21, and 28 or at any other day (unscheduled visits) when patients felt unwell. Parents/guardians were informed and encouraged to bring their children to the clinic whenever they were unwell without waiting for scheduled visits. Patients who could not come for their scheduled visit by mid-day were visited at home by a member of the study team and asked to come to the health centre. In case a patient travelled to other places and could not be traced for scheduled follow-up, he/she was classified as lost to follow-up and was withdrawn from the study. During the visits, clinical and safety assessments were performed, axillary temperature was measured, and a blood slide for parasite count was taken. On day 7, 14, and 28, and dried blood spots (DBS) were collected on filter papers for genotyping, Additional file 2: Table S1.

### Sample collection and examination

Samples were collected through a finger prick for collection of blood sample for malaria rapid diagnostic test (RDT) and collection of thick and thin blood smears for detection of malaria parasites by microscopy. From each patient, dried blood spots (DBS) on Whatman III filter papers were collected for laboratory analysis of malaria parasites, including *P. falciparum* diversity, molecular markers of anti-malarial resistance and distinguishing recrudescent from new infections by PCR genotyping. Collected blood slides were stained with 3% Giemsa for 30–45 min and examined by microscopy to detect presence of malaria parasites and the level of parasitaemia, *Plasmodium* species, and presence of gametocytes. Parasitaemia was measured by counting the number of asexual parasites against 200 white blood cells (WBCs) in thick blood films and detection of the different parasite species was done on thin films as previously described [19]. Parasite density, expressed as the number of asexual parasites per µl of blood, was calculated by dividing the number of asexual parasites by the number of white blood cells counted and then multiplying by an assumed white blood cell density (typically 6000 per µl) [19]. A blood slide was declared negative when examination of 100 high power fields did not reveal the presence of any malaria parasite. For quality control, each slide was re-examined by a second microscopist and those with discrepancy were re-examined by the third microscopist. Further disagreement was resolved by a team of three microscopists who examined the same slide at the same time. Final parasitaemia was calculated as the average between the two closest readings.

### Sample processing, parasite genotyping and molecular analysis

Parasite deoxyribonucleic acid (DNA) was extracted from DBS using QIAamp DNA blood mid kit (Qiagen GmbH, Hilden, Germany) according to the manufacturer’s instructions. Genotype analysis was conducted in order to differentiate a recrudescent (treatment failure/same parasite strain) from a newly acquired infection (reinfection/different parasite strain) among recurrent parasitemia found during follow-up. This analysis was based on the extensive diversity in the following *P. falciparum* genes: merozoite surface proteins 1 and 2 (*msp1* and *msp2*), and glutamate rich protein (*glurp*) genes.

Distinguishing recrudescent from new infections was done by genotyping three length-polymorphic genetic markers (*msp1* and *msp2*, and *glurp)* using gel electrophoresis detection based on the WHO protocol [23, 24]. Recrudescence was determined by comparing samples that contained at least one matching allelic band, indicating fragment size similarities qualifying as a match for each marker, with a difference of 10 bp for *msp1* and *msp2* and 50 bp for *glurp*, from paired samples collected on day of enrolment (D0) and day of recurrent infection (R0). A reinfection was defined as the absence of any matching allelic band in at least one marker in the paired blood samples. *msp2* was run for all samples, then *glurp* for those matching at *msp2*, and *msp1* for those matching at both msp2 and *glurp*. The classification of a recrudescence was based on 3/3 algorithm, i,e match when all markers had genotyping results and in case of non-determinant results in any marker, results for available markers were used to assess [20]. The presence of recrudescence was assessed using a recent 2/3 algorithm as recommended by the WHO in 2021 [20]. The genotyping data used for molecular correction has been included as a supplemental file (Additional file 1: Table S2).

### Outcome classification

The primary end point was parasitological cure on day 28 as per WHO protocol of 2009 [15, 24] while secondary end points included occurrence and severity of adverse events. Treatment outcomes were classified as (1) Early treatment failure (ETF) if the patient had presence of parasitaemia and danger signs on day 1, 2 and 3 or persistence of parasitaemia till day 3. (2) Late clinical failure (LCF) was defined as presence of danger signs with parasitaemia between day 4 and 28 to a patient who didn’t qualify as early treatment failure. (3) Late parasitological failure (LPF); a patient who had parasitaemia between day 7 and 28 and was not classified as early treatment failure. (4) Adequate clinical and parasitological response (ACPR) was defined as absence of parasitaemia to a patient who wasn’t classified as early, late clinical, or late parasitological failure. (5) Lost to follow-up occurred when despite all reasonable efforts, an enrolled patient does not attend the scheduled visits and cannot be found, the patient was withdrawn from the study. (6) Withdrawal; due to consent withdrawal, failure to complete treatment and protocol violation [22].

### Ethical considerations

Ethical clearance was obtained from the Medical Research Coordinating Committee (MRCC) of the National Institute for Medical Research with number NIMR/HQ/R.8a/Vol.IX/2687. Permission to conduct the study at the health facilities was sought in writing from the relevant regional and district medical authorities. Oral and written informed consent was obtained from parents or guardians of all eligible patients before they were screened for possible inclusion into the study.

### Data management and analysis

The first data entry was performed at the study sites and followed by second data entry, which was centrally done at Muhimbili University of Health and Allied Sciences (MUHAS) after the end of data collection. The data was entered into a Microsoft Access database, and later validated, cleaned, and analysed using STATA for Windows, version 13 (STATA Corporation, TX-USA). Continuous variables were summarized using mean, median, standard deviation, and interquartile range while categorical variables were summarized using percentages. Treatment outcomes (primary and secondary) outcomes were analysed based on the WHO protocol and presented by site. Kaplan–Meier analysis (curves) were used to present the time to parasite infections based on PCR un-corrected and corrected. Lost to follow-up, withdrawal, protocol violation and individuals whose PCR results could not be resolved were reported as non-determined were excluded from the analysis of PCR-corrected treatment outcome. While, individuals with reinfection, lost to follow-up and withdrawal were censored in KM analysis.

## Results

### Baseline characteristics, enrolment and follow-up

During the study period, 653 children were screened for eligibility and 349 (53.7%) were enrolled between April and August 2018. Of the enrolled children, 345 (98.9%) completed 28 days follow-up or attained the treatment outcomes (Table 1).

**Table 1:** Baseline characteristics of enrolled patients in all four sites

Characteristics	Kibaha (n = 85)	Mlimba (n = 88)	Mkuzi (n = 88)	Ujiji (n = 88)
Males, n (%)	47 (55.3)	40 (45.5)	55 (62.5)	47 (53.4)
Age (Years)				
Median (IQR)	6.5 (4.6–8.0)	4.6 (2.5 –6.7)	6.0 (3.8 –8.2)	3.1 (2.0–6.3)
Weight (kgs)				
Mean (95%CI)	19.6 (18.4–20.7)	17.1 (15.9–18.7)	18.3 (17.1–19.4)	13.9 (12.8–15.0)
Height (cm)				
Mean (95%CI)	113.2 (109.6–116.9)	100.4 (96.4 –104.5)	111.0 (107.1–114.9)	95.7 (91.6–99.8)
Temp (°C)				
Mean (SD)	38.3 (1.3)	38.2 (1.1)	38.2 (1.3)	37.9 (1.3)
Parasitemia (µl)				
Geometric Mean (95%CI)	17,966 (12,503–25,816)	24,038 (17,461–33,750)	31,507 (24,016–41,333)	29,019 (20,636–40,806)

*°C* degree Celsius, *Kg* Kilogram, *SD* standard deviation, *95% CI* 95% confidence interval, *n* number of patients, *IQR* Inter quartile range

**Fig. 2:**
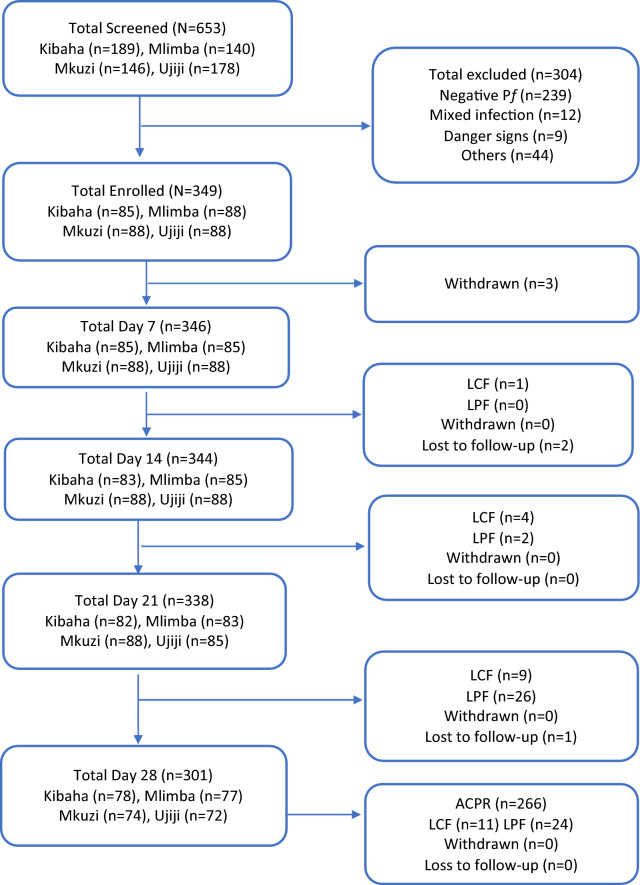
Flow chart of study patients. *ACPR* adequate clinical and parasitological response, *LCF* late clinical failure, *LPF* late parasitological failure, *p.f* plasmodium falciparum

During screening, we excluded 304 children mainly because of presence of fever due to other causes with negative results by mRDTs or microscopy, low parasitemia outside the defined range, severe malaria or living outside the study area (Fig. 2). Table 1 summarizes the demographic and laboratory baseline data of the participants. With the exception of the Kibaha site, which enrolled 85 patients due to low malaria transmission, all the other sites recruited 88 children. Ujiji site recruited children with lower age, body weight and height compared to other sites. Mkuzi site recruited more female patients compared to other sites. The average axillary temperature was similar at all sites. The geometric mean parasite density (parasitemia per μL) ranged from 17,966 at Kibaha site to 31,507 at Mkuzi site, (Table 1).

### Efficacy outcomes

The treatment outcomes are summarized in Table 2. As per-Kaplan Meier; PCR uncorrected adequate cure rate was 64.8% 95% CI (53.8–73.7) in Mkuzi, 73.9% (63.3–81.8) in Ujiji and over 85%) in Kibaha and Mlimba sites. PCR-corrected cures rate (Kaplan–Meier) was 97.6% 95% CI (90.5–99.4) in Kibaha, 100% in Mlimba, 98.6% (90.7–99.8) in Mkuzi and 98.8% (91.9–99.8) in Ujiji. One patient from Mlimba was withdrawn after enrollment because of protocol violation, i.e., the patient had features of severe malaria (severe anaemia) at enrolment. Hence, according to the study protocol, the patient was excluded. After intake of the first dose of AL, the patient developed features of severe malaria and rescue treatment with injectable artesunate was given. The PCR results for three patients (one in Mlimba, one in Mkuzi, and one in Ujiji) with recurrent parasitaemia were not determined by PCR methods. These cases were withdrawn from the per-protocol analysis or censored in KM analysis as were the patients with reinfections, loss to follow up or protocol violation. Using the newly recommended PCR -corrected cure rates based on 2/3 algorithm as a sensitivity analysis, four more recrudescent infections were detected, Additional file 1: Table S2.

**Table 2:** Parasitological and clinical outcomes of enrolled patients

Outcome	Kibaha (n = 83)		Mlimba (n = 85)		Mkuzi (n = 88)		Ujiji (n = 88)	
	n (%)	95%CI	n (%)	95%CI	n (%)	95%CI	n (%)	95%CI
PCR Uncorrected								
ETF	0	–	0	–	0	–	0	–
LCF	8 (9.6)	4.9–18.2	3 (3.5)	1.1–10.5	8 (9.1)	4.6–17.2	6 (6.8)	3.1–14.5
LPF	4 (4.8)	1.8–12.2	9 (10.6)	5.6–19.2	23 (26.1)	18–36.4	17 (19.3)	12.3–29
ACPR	71 (85.5)	76.1–91.6	73 (85.9)	76.7–91.8	57 (64.8)	54.2–74.1	65 (73.9)	63.6–82
Total	83		85		88		88	
Excluded								
Withdrawn/lost	2		3		0		0	
Cumulative cure rate	85.6	76.0–91.5	85.9	76.5–91.7	64.8	53.8–73.7	73.9	63.3–81.8
PCR Corrected								
ETF	0	–	0	–	0	–	0	–
LCF	1 (1.4)	0.2–9.4	0	–	0	–	0	–
LPF	1(1.4)	–	0	–	1 (1.7)	0.2–11.5	1 (1.5)	0.2–10.2
ACPR	71 (97.3)	90.6–99.8	73 (100)	–	57 (98.3)	88.5–99.8	65 (98.5)	89.8–99.8
Total	73		73		58		66	
Excluded								
Reinfections	10		11		28		21	
PCR unknown	0		1		1		1	
Missing PCR	0		0		1		0	
Withdrawn/lost	2		3		0		0	
Cumulative cure rate (Kaplan–Meier)	97.6	90.5–99.4	100	-	98.6	90.7–99.8	98.8	91.9–99.8

*ETF* Early Treatment Failure, *LPF* Late Parasitological Response, *LCF* Late Clinical Failure, *ACPR* Adequate Clinical and Parasitological Response

### Parasite clearance

No patient had parasitaemia on day 3; however, on day 2 the parasite rates ranged from 5.9% in Mlimba to 22.7% in Mkuzi sites (Table 3).

**Table 3:** Day 3 positivity rates among patients enrolled at the four sites

Follow-up day	Kibaha (n = 85)(%)	Mlimba (n = 85*)(%)	Mkuzi (n = 88) (%)	Ujiji (n = 88) (%)	Total (N = 346) (%)
Day 1	63 (74.1)	70(82.3)	77(87.5)	74(84.1)	228(83.8)
Day 2	7(8.23)	5(5.9)	20(22.7)	7(7.9)	39(14.7)
Day 3	0(0)	0(0)	0(0)	0(0)	0(0)

^*^2 patients were withdrawn from the study

### Safety outcomes

Fifty-eight (16.3%) patients experienced at least one adverse event (AE), of which 17 had two adverse events and three patients experienced three adverse events The most common events were cough 32 (42.0%), running nose and abdominal pain each in 9 (11.5%), and fever in 8 (10.3%) of the events. No serious adverse events were reported. Distribution of events by study sites is shown in Table 4.

**Table 4:** Reported adverse events by site

Adverse event	Study site				Total
	Kibaha	Mlimba	Mkuzi	Ujiji	
Cough	5 (23.81)	3 (27.27)	13 (76.47)	11 (37.93)	32 (41.02)
Abdominal pain	6 (28.57)	2 (18.18)	1 (5.88)	0 (0)	9 (11.54)
Running nose	4 (19.05)	2 (18.18)	0 (0)	3 (10.34)	9 (11.54)
Fever	3 (14.29)	2 (18.18)	0 (0)	3 (10.34)	8 (10.26)
Vomiting	1 (4.76)	0 (0)	0 (0)	6 (20.69)	7 (8.97)
Diarrhoea	0 (0)	1 (9.09)	2 (11.76)	3 (10.34)	6 (7.69)
Painful micturition	2 (9.52)	0 (0)	0 (0)	3 (10.34)	5 (6.41)
Anal itching	0 (0)	0 (0)	1 (5.88)	0 (0)	1 (1.28)
Anaemia	0 (0)	1 (9.09)	0 (0)	0 (0)	1 (1.28)
Total	21 (100)	11 (100)	17 (100)	29 (100)	78 (100)

thus confirming the results obtained from previous studies.

## Discussion

The findings indicate that AL, the recommended first-line treatment for uncomplicated falciparum malaria in Tanzania, is still efficacious in these study sites, with a PCR-corrected cure rate of > 97% for both per protocol analysis and survival analysis, thus confirming the results obtained from the previous studies [16, 17, 21]. The PCR corrected cure rates were similar to those observed in previous studies conducted in 2011 and 2016 [16, 19]. Furthermore, PCR cure rates presented using the 2/3 algorithm were reduced at Kibaha (93.8%) as more recrudescent infections were detected. Previous reports show particular concern that the 3/3 algorithm is likely to have underestimated the true failure rate by approximately a twofold factor [20]

The risk of recurrent parasitemia was high in Mkuzi and Ujiji, similar to the previous study that was conducted in 2014 [19]. Some of contributing factors could be sustained high transmission and reduced prophylactic effect after treatment with AL, which is known to have short protective effects attributed to short elimination half-life of lumefantrine compared to other partner drugs [22].

The absorption of lumefantrine is known to have a high variability, and suboptimal drug levels could potentially result from inadequate concomitant fat intake [7, 9]. In this study, caregivers were encouraged to accompany each AL dose with milk or fat-containing food, but this could not be confirmed by study staff.

Widescale use of AL treatment has been associated with selection of wild type alleles (*Pfmdr1 N86* and *Pfcrt K76*). Nonetheless, the presence of mutations does not always correlate with the measured cure rate [16, 23]. Hence molecular analysis for *Pfmdr* and *Pfcrt* mutation together with *K-13* mutations may shade the picture on the underlying causes of tolerance/reduced efficacy. The development of artemisinin resistance was first observed in South East Asia and subsequently confirmed by a molecular marker [13, 24]. It has now been detected in several sub-Saharan African countries, including Tanzania, Kenya, Uganda, Eritrea, and Rwanda [14, 25, 26] and from Kagera region (Ishengoma DS, unpublished data). Countering onset of resistance might need deliberate strategies aimed at slowing the reduction in ACT effectiveness [26]. Can the treatment and cure of as many people as possible be achieved without significantly promoting drug resistance? The recently released WHO document on strategy to combat anti-malarial drug resistance recommends a number of interventions including optimizing use of diagnostics and diversifying ACT markets in countries to reduce drug pressure [26].

This study also showed that AL was well tolerated with minimal adverse events (AEs). The common adverse events were cough, runny nose, abdominal pain, and fever, which were similar to other studies conducted in Tanzania [16, 17]. Uwimana et al. [14], Kakolwa et al. [17, 20, 21] reported a similar safety profile of AL when used for the treatment of uncomplicated falciparum malaria. No serious adverse were reported in this study. Thorough clinical and laboratory assessment, including evaluation of haemoglobin levels in suspected cases, and monitoring of patients with high parasitaemia (> 100,000 asexual parasites/µl) prevented inclusion of patients with suspected severe malaria or other disease conditions.

## Conclusion

The observed high cure rates of ≥ 97% and high safety profile at all study sites suggests that AL is still efficacious and safe in these study sites after its widescale use in Tanzania. Nevertheless, the significant occurrences of high recurrences (< 74% PCR uncorrected treatment failure) in Mkuzi and Ujiji, along with recent reports indicating emergence of partial artemisinin resistance in SSA, including Tanzania, raise concerns about its long-term viability as a treatment option in Africa. Consequently, there is a need to explore new strategies for the treatment of uncomplicated malaria.

### Supplementary Information

**Additional file 1. **TES 2018 PCR data.

**Additional file 2: Table S1. **Clinical and Laboratory assessments during follow-up visits.

## Data Availability

Data will be available upon request to the corresponding author.
