# Supplementary material for: Efficacy and safety of artemether-lumefantrine for the treatment of uncomplicated falciparum malaria in mainland Tanzania, 2018
Source: Malar J. 2024 Apr 6;23:95. doi: 10.1186/s12936-024-04926-x (PMC10998292; doi:10.1186/s12936-024-04926-x)
Supplement: Supplementary file 2 — Additional file 2: Table S1. Clinical and Laboratory assessments during follow-up visits. [file 12936_2024_4926_MOESM2_ESM.docx]

**Additional file 2: Table S1: Clinical and Laboratory assessments during follow-up visits**

| **Procedures** | **Visits** | | | | | | | | |
| --- | --- | --- | --- | --- | --- | --- | --- | --- | --- |
|  | **0** | **1** | **2** | **3** | **7** | **14** | **21** | **28** | **Other day** |
| Clinical Assessment | **x** | **x** | **x** | **x** | **x** | **x** | **x** | **x** | **(x)** |
| Blood slide for parasite count | **x** | **x** | **x** | **x** | **x** | **x** | **x** | **x** | **(x)** |
| Blood for genotyping | **X** |  |  |  | **X** | **x** | **X** | **X** | **(x)** |
| Blood for haemoglobin | **X** |  |  |  | **(X)** | **(X)** | **(X)** | **(X)** | **(x)** |
| Blood for molecular markers | **x** |  |  |  | **x** | **x** | **x** | **x** | **(x)** |

Parentheses denote conditional or optional activities.
